# Preparation and Electromagnetic Interference Shielding Performance of TPU/MWCNT/BiFeO_3_ Composites

**DOI:** 10.3390/polym18182214

**Published:** 2026-09-11

**Authors:** Tie Geng, Junhao Tang, Chenhao Xu, Shaobin Cai, Xinchao Wang, Xiaoli Bai, Jiayu Liao, Tongfei Zhang, Baichuan He, Pengyu He, Mengling Li

**Affiliations:** Henan Key Laboratory of Superhard Abrasives and Grinding Equipment, School of Mechanical and Electrical Engineering, Henan University of Technology, No. 100, Lianhua Street, National High-Tech Development Zone, Zhengzhou 450001, China

**Keywords:** thermoplastic polyurethane, multi-walled carbon nanotubes, bismuth ferrite, electromagnetic shielding

## Abstract

The rapid advancement of information technology and pervasive use of electronic devices has exacerbated electromagnetic radiation pollution and interference, driving the demand for lightweight, flexible, and high-efficiency electromagnetic shielding materials in materials research. As a high-performance elastomer, thermoplastic polyurethane (TPU) possesses excellent elasticity, wear resistance, oil resistance and processability, making it promising for flexible electronics and wearable devices. However, pure TPU is electrically insulating and exhibits nearly no electromagnetic shielding capability, which requires conductive filler incorporation for functional modification. Herein, ternary TPU/MWCNT/BiFeO_3_ composites were fabricated via solution blending and hot pressing, using multi-walled carbon nanotubes (MWCNTs) and bismuth ferrite (BiFeO_3_) as conductive and dielectric fillers within the TPU matrix. The effects of filler content on the microstructure, thermal stability, mechanical properties and electromagnetic shielding performance of composites, together with the relevant mechanisms, were systematically studied. For the ternary TPU/MWCNT/BiFeO_3_ system, the introduction of BiFeO_3_ continuously increases the char residue rate of the composites to 16.01%, while accelerating the reaction process during the main thermal decomposition stage. The mechanical properties gradually deteriorate with the increase in BiFeO_3_ content, and the composite with 5 wt% BiFeO_3_ almost loses its elastomeric characteristics. The electromagnetic shielding effectiveness (SE) presents a trend of initial increase and subsequent decrease. The composite with 3 wt% BiFeO_3_ exhibits the optimal shielding performance, with a 24.7% enhancement in total SE compared with the reference TPU/MWCNT composite containing 1% MWCNT. This improvement is attributed to the interfacial polarization and dipole polarization induced by the appropriate amount of BiFeO_3_, which effectively strengthen the electromagnetic wave absorption loss capacity of the composites.

## 1. Introduction

Numerous studies have systematically investigated the effects of varying MWCNT loadings on the microstructure, mechanical properties and electromagnetic interference shielding effectiveness of binary TPU/MWCNT composites [1,2,3]. Relevant results demonstrate that as MWCNT loading increases, continuous and intact conductive networks are gradually constructed within the matrix, accompanied by a simultaneous improvement in the overall shielding effectiveness of the materials [4]. Once the MWCNT loading exceeds the percolation threshold, the composites deliver outstanding shielding performance across the Ku band (12.4–18.0 GHz), which fully verifies that conductive loss acts as the dominant mechanism responsible for electromagnetic wave attenuation in carbon nanotube-based composite systems [5]. Nevertheless, binary composites solely filled with MWCNTs possess obvious drawbacks. Their shielding performance heavily relies on conductive loss while contributing limited dielectric absorption loss to incident electromagnetic waves, leaving considerable room for the optimization of wave absorption capacity [6,7]. Meanwhile, high MWCNT loadings tend to induce severe filler agglomeration, drastically deteriorating mechanical indicators such as tensile strength and toughness of composites, elevating melt viscosity, and increasing the processing difficulty during molding [8].

Based on the above considerations, ternary TPU/MWCNT/BiFeO_3_ composites were fabricated in this chapter by incorporating bismuth ferrite (BiFeO_3_) particles with dielectric loss characteristics while fixing the MWCNT loading at 1 wt%. The selection of 1 wt% MWCNT as the fixed content is mainly based on the following reasons: at this loading, MWCNTs have constructed preliminary conductive networks within the TPU matrix, which can provide fundamental channels for conductive loss [9]. Meanwhile, sufficient interfacial space and room for synergistic effects are reserved for the incorporation of BiFeO_3_, facilitating the investigation of the influence of the second-phase filler on the overall performance of the composites.

As a room-temperature multiferroic material, bismuth ferrite (BiFeO_3_) has attracted extensive attention in the field of electromagnetic functional materials in recent years [10]. Its unique perovskite crystal structure endows BiFeO_3_ with simultaneous ferroelectric and antiferromagnetic ordering characteristics, delivering distinct dielectric relaxation behavior and desirable magnetic loss capacity in the microwave frequency band [11,12]. In terms of the loss mechanism, the dielectric loss of BiFeO_3_ primarily originates from the relaxation processes induced by oxygen vacancies, ferroelectric domain wall movement, and dipole polarization, which enable its great potential as an electromagnetic wave absorbing material [13]. Previous studies have demonstrated that BiFeO_3_ exhibits a high dielectric loss value within the frequency range of 12.4–18 GHz and possesses thermally stable dielectric properties, rendering it promising for high-temperature microwave absorption applications [14,15].

In polymer-based electromagnetic interference (EMI) shielding composites, the filler type, intrinsic electromagnetic properties, and dispersion state within the matrix directly determine the conductive, dielectric and magnetic loss behaviors of composites, and further govern their overall shielding effectiveness and comprehensive mechanical performance [16,17]. Carbon-based fillers (carbon nanotubes, graphene, carbon black, etc.) are the most widely used conductive dispersed fillers. Among them, multi-walled carbon nanotubes (MWCNTs) can build continuous conductive networks at a low percolation threshold owing to their high aspect ratio. Electromagnetic waves are attenuated through ohmic loss, and MWCNTs possess the advantages of light weight and excellent electrical conductivity [18]. However, shielding systems filled solely with carbon-based fillers are dominated by conductive loss. Severe impedance mismatch causes a large fraction of electromagnetic waves to be directly reflected at the material surface, leading to a low proportion of internal absorption. Increasing filler loading to improve shielding performance will in turn trigger filler agglomeration and result in a substantial deterioration in matrix toughness.

Magnetic-dielectric ceramic fillers (e.g., bismuth ferrite, ferrites) represent another important category of functional fillers. They dissipate electromagnetic wave energy mainly via dipole polarization, interfacial polarization, hysteresis loss and eddy-current loss, which helps optimize impedance matching and raise the proportion of internal electromagnetic-wave absorption [18]. Nevertheless, such ceramic fillers exhibit poor intrinsic electrical conductivity. When incorporated into polymers alone, they can hardly construct conductive pathways, resulting in limited overall shielding effectiveness. Meanwhile, inorganic ceramic particles show weak compatibility with polymer matrices, and high-loading conditions also degrade the elasticity and tensile properties of materials.

Combining carbon-based conductive fillers with magnetic-dielectric ceramic fillers is an effective strategy to realize conductive loss, dielectric loss and magnetic loss simultaneously. Benefiting from the complementary advantages of the two types of fillers, a synergistic multi-loss mechanism of “conductive-dielectric-magnetic” can be constructed at a relatively low total filler loading, which is expected to increase the proportion of electromagnetic-wave absorption without excessively raising the filler content. Nevertheless, hybrid filler systems still face considerable challenges. The coexistence of multiple fillers aggravates the risk of agglomeration, and the filler-matrix interface structure becomes complicated. The filler ratio and dispersion process exert strong coupling effects on the micromorphology, mechanical properties, thermal properties and electromagnetic performance of composites [16,17].

In this experiment, ternary TPU/MWCNT/BiFeO_3_ composites with a fixed MWCNT loading of 1 wt% and varied BiFeO_3_ contents were fabricated via a solution blending method. The effects of BiFeO_3_ dosage on the microstructure, thermal stability, mechanical properties, and electromagnetic shielding effectiveness of the composites were systematically investigated. The synergistic mechanism between conductive fillers and dielectric fillers was analyzed, and the electromagnetic wave attenuation law under the dual conductive loss–dielectric loss mechanism was comprehensively explored [18].

## 2. Mechanism of Electromagnetic Interference (EMI) Shielding

Electromagnetic interference (EMI) shielding refers to a technical method that attenuates electromagnetic waves via conductive or magnetic materials, so as to block electromagnetic radiation from entering or escaping a specific area. According to Schelkunoff’s EMI shielding theory, three primary processes occur when electromagnetic waves impinge on the surface of shielding materials, namely reflection loss, absorption loss and multiple reflection loss.

Reflection loss (SER) represents the energy loss originating from the reflection of electromagnetic waves at the material surface due to impedance mismatch. When incident electromagnetic waves propagate from air (impedance Z0 ≈ 377 Ω) into the shielding material (impedance Zm), partial waves are reflected back into the air medium owing to the impedance difference between the two media. The magnitude of reflection loss depends on the electrical conductivity (σ) and magnetic permeability (μ) of materials, which can be expressed by the following equation.

(1) $$\begin{array}{c} SER=20\log_{10}(\frac{Z_{m}}{{4Z}_{0}})=20\log_{10}(\frac{1}{4}\sqrt{\frac{\sigma }{\pi f\mu }}) \end{array}$$

where f denotes the frequency of electromagnetic waves. It can be observed from the formula that higher electrical conductivity and magnetic permeability of the material lead to more remarkable reflection loss.

Absorption loss (SEA) refers to the energy loss when electromagnetic waves penetrate the interior of materials and are attenuated and dissipated. As electromagnetic waves propagate inside the material, free electrons in conductive fillers generate ohmic loss under the action of electromagnetic fields; magnetic domains of magnetic fillers produce magnetic loss in alternating magnetic fields; dipoles of dielectric fillers undergo orientation polarization under alternating electric fields to induce dielectric loss. The magnitude of absorption loss is closely correlated with the electrical conductivity, magnetic permeability, dielectric constant and thickness of the material:

(2) $$\mathrm{SEA}\;=\;20\log_{10}(e^{t/\delta })\;=\;8.686\frac{t}{\delta }$$

where t represents the material thickness, and δ denotes the skin depth, which follows the equation δ = (πfμσ) − 1/2. The absorption loss increases with rising frequency and elevated electrical conductivity.

**Figure 1:**
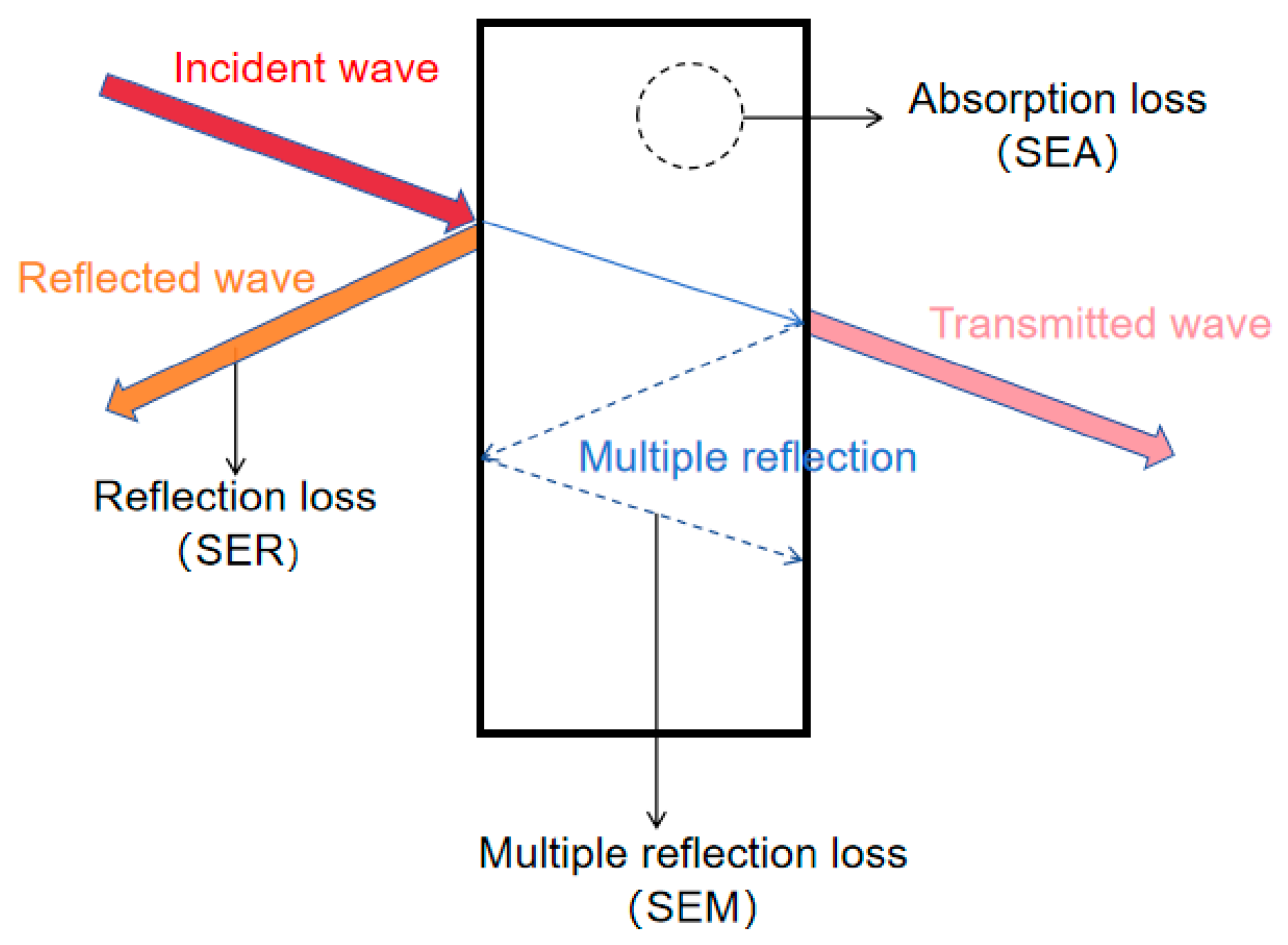
Schematic illustration of EMI shielding mechanism.

Figure 1 is the schematic of electromagnetic interference shielding mechanism. Multiple reflection loss (SEM) corresponds to the energy loss generated by repeated reflection and transmission of electromagnetic waves inside materials. After electromagnetic waves penetrate the first interface, multiple reflections occur within the material or at interlayer interfaces, which extend the interaction duration between electromagnetic waves and the material and thus strengthen the attenuation effect. When the material thickness is much larger than the skin depth, the multiple reflection loss can be neglected.

The electromagnetic interference (EMI) shielding effectiveness of the materials was tested using a Ceyear 3672D vector network analyzer manufactured by Beijing Oriental Zhongke Integration Technology Co., Ltd. (Beijing, China). The vector network analyzer evaluates the electromagnetic interference (EMI) shielding performance of materials by measuring the transmission coefficient (S21) and reflection coefficient (S11) with and without the sample. S21 represents the coefficient for electromagnetic-wave transmission from port 1 to port 2, which reflects the wave-transmission capability of the material. S11 is the reflection coefficient at port 1 and characterizes the electromagnetic-wave reflection capability of the material surface. Based on the measured S-parameters, the total shielding effectiveness (SET), absorption-related shielding loss (SEA) and reflection-related shielding loss (SER) of the material can be calculated. Generally, the power reflection coefficient is defined as R = |S11|^2^, which represents the proportion of incident electromagnetic wave power reflected by the material. The power transmission coefficient T is given by T = |S21|^2^, standing for the fraction of power transmitted through the material. The absorption coefficient A corresponds to the fraction of power absorbed by the material and satisfies the relation A = 1 − R − T. These coefficients describe the power distribution of electromagnetic waves interacting with the material.

The formula for total shielding effectiveness is given by:

(3) $$\begin{array}{c} SET\;=\;-10lg{|S_{21}|}^{2\;}=\;-20lg|S_{21}| \end{array}$$

The calculation formula for SER is as follows:

(4) $$\begin{array}{c} SER\;=\;-10lg(1\;-\;{|S_{11}|}^{2}) \end{array}$$

The formula for absorption-related shielding loss is given by:

(5) $$\begin{array}{c} SEA\;=\;SET\;-\;SER\;-\;SEM \end{array}$$

where SEM is the correction term for multiple-reflection loss. For EMI shielding, the contribution of multiple-reflection loss to the overall shielding performance is relatively weak and can be neglected in calculations. Under this circumstance, SEA ≈ SET − SER.

It should be noted that a distinction should be made between shielding-loss components SEA, SER and power coefficients A, R, T. SEA and SER are in decibels (dB), which characterize the shielding attenuation contributed by internal absorption inside the material and interfacial reflection, respectively. In comparison, A, R and T are dimensionless power fractions satisfying A + R + T = 1, which correspond to the fractions of incident electromagnetic power that are absorbed, reflected and transmitted through the material. SEA is not equivalent to the absorption coefficient A: SEA describes the attenuation degree of electromagnetic waves after entering the material interior, while A stands for the final proportion of total incident power genuinely absorbed by the composite. Multiple reflections generated by internal interfaces within the material cause part of electromagnetic waves to escape from the sample surface. Therefore, a higher SEA does not guarantee a larger A value. This concept is essential for understanding the EMI shielding mechanism.

## 3. Preparation and Process Route of TPU/MWCNT/BiFeO_3_ Composites

### 3.1. Experimental Materials

The raw materials adopted in this work are listed in Table 1.

### 3.2. Experimental Equipment

The experimental instruments and equipment are listed in Table 2.

### 3.3. Component Ratios of TPU/MWCNT/BiFeO_3_ Composite Materials

TPU/MWCNT/BiFeO_3_ composites with a fixed MWCNT mass fraction of 1 wt%, and BiFeO_3_ mass fractions of 0 wt%, 1 wt%, 3 wt%, and 5 wt%, respectively.

### 3.4. Fabrication Process of TPU/MWCNT/BiFeO_3_ Composites

First, TPU pellets, MWCNT powder and BiFeO_3_ powder were separately placed in a vacuum drying oven and dried at 100 °C for 6 h to remove moisture. Glassware such as beakers and Petri dishes used in the experiment were cleaned and dried for later use.

During sample fabrication, TPU pellets (45 g) were weighed into a dry beaker according to the designed component ratios. MWCNT powder (fixed at 1 wt% relative to the mass of TPU, 0.45 g) and BiFeO_3_ powder with varied loadings (1 wt%, 3 wt% and 5 wt% relative to TPU mass, corresponding to 0.45 g, 1.35 g and 2.25 g, respectively) were weighed using weighing papers, followed by measuring 200 mL of DMF solvent. The MWCNT powder was first added to DMF and homogeneously dispersed via a high-speed stirrer. Afterwards, BiFeO_3_ powder with the corresponding mass fraction was introduced into the MWCNT dispersion, and stirring was continued to achieve uniform mixing. Finally, TPU pellets were incorporated, and stirring was maintained until TPU was completely dissolved to obtain a homogeneous TPU/MWCNT/BiFeO_3_/DMF mixed solution.

The mixed solution was poured into Petri dishes and vacuum-dried at 140 °C in a vacuum drying oven to evaporate the DMF solvent, yielding TPU/MWCNT/BiFeO_3_ composites. The as-prepared materials were taken out and cut into small pieces. A thin layer of release agent was evenly sprayed onto the mold surface of a plate vulcanizer, followed by standing for a period until the release agent dried completely. Subsequently, the cut materials were loaded into the mold and placed on the plate vulcanizer for pre-melting at 185 °C for 15 min [19]. Afterwards, the temperature was kept constant, and a pressure of 8 MPa was applied and maintained for 20 min. After the pressure-holding stage, the sample was cooled to room temperature under constant pressure before demolding, and TPU/MWCNT/BiFeO_3_ composite specimens were finally obtained [20].

**Figure 2:**
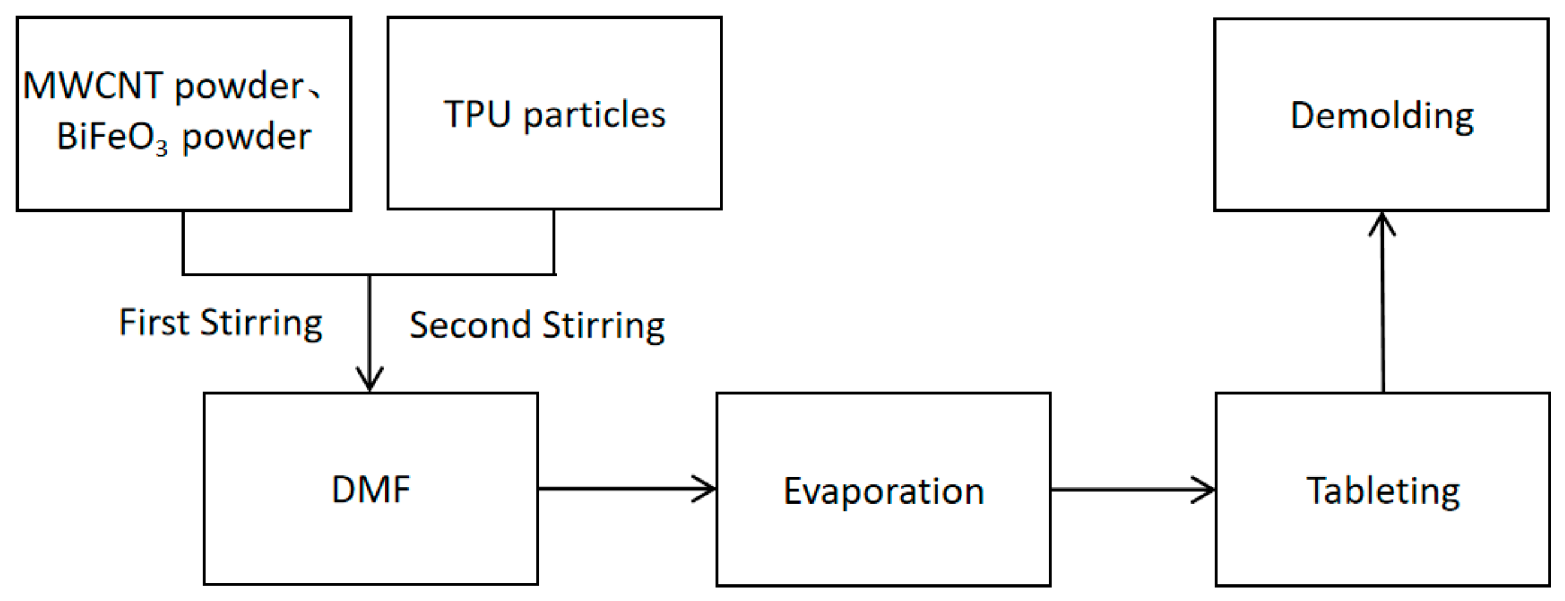
Fabrication process of TPU/MWCNT/BiFeO_3_ composites.

By fixing the MWCNT loading at 1 wt% and varying the addition amount of BiFeO_3_, TPU/MWCNT/BiFeO_3_ composites with BiFeO_3_ mass fractions of 1 wt%, 3 wt% and 5 wt% were fabricated, respectively. The fabrication procedure is illustrated in Figure 2.

## 4. Results and Discussion

### 4.1. X-Ray Diffraction (XRD) Analysis of TPU/MWCNT/BiFeO_3_ Composites

**Figure 3:**
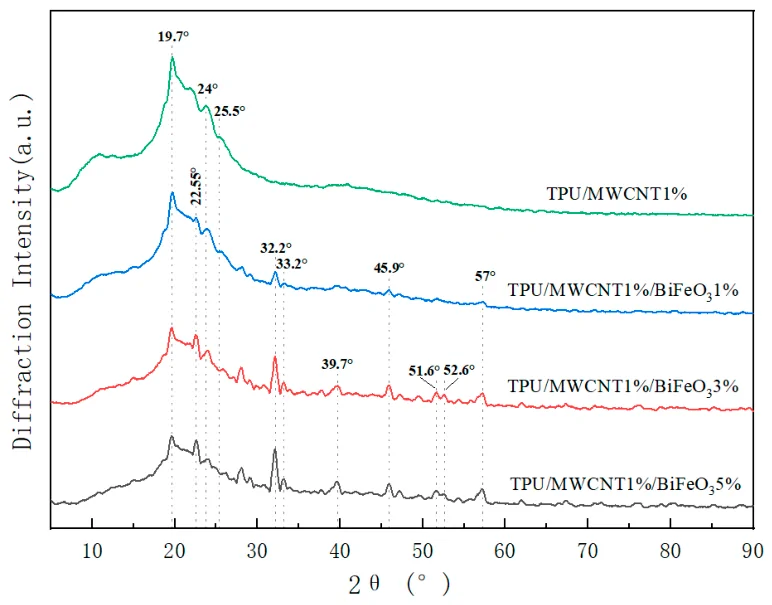
X-ray diffraction (XRD) patterns of TPU/MWCNT/BiFeO_3_ composites.

The crystal structure of BiFeO_3_ and its dispersion state in the matrix were confirmed by analyzing the X-ray diffraction (XRD) patterns of TPU/MWCNT/BiFeO_3_ composites (Figure 3).

The XRD results show that the TPU/MWCNT/BiFeO_3_ composite with 1 wt% BiFeO_3_ exhibits characteristic diffraction peaks of BiFeO_3_ at 2θ of 22.5°, 32.2°, 33.2°, 45.9°, and 57°. For the TPU/MWCNT/BiFeO_3_ composites with 3 wt% and 5 wt% BiFeO_3_, additional characteristic peaks of BiFeO_3_ appear at 2θ of 39.7°, 51.6°, and 52.6°. Specifically, the diffraction peak at 22.5° corresponds to the (012) crystal plane, the peak at 32.2° corresponds to the (104) crystal plane, and the peak at 33.2° corresponds to the (110) crystal plane of BiFeO_3_. The diffraction peak at 39.7° is assigned to the (006) or (202) crystal plane, the peak at 45.9° to the (024) crystal plane, the peak at 51.6° to the (116) crystal plane, the peak at 52.6° to the (018) crystal plane, and the peak at 57° to the (122) or (018) crystal plane of BiFeO_3_. The appearance of these typical BiFeO_3_ diffraction peaks verifies the successful fabrication of target composites. With the increase in BiFeO_3_ content from 1 wt% to 5 wt%, the intensity of BiFeO_3_ characteristic peaks gradually increases, indicating the simultaneous improvement in crystallinity and loading of BiFeO_3_. Notably, the main diffraction peak at 32.2° shows the most significant intensity enhancement, suggesting the possible preferred orientation of the (104)/(110) crystal planes.

The coexistence of shoulder peaks at 2θ = 24° and 2θ = 25.5° in the 1 wt% BiFeO_3_ composite demonstrates the uniform dispersion of MWCNTs in the TPU matrix. As the BiFeO_3_ content increases, the intensity of the peak at 24° decreases, while the shoulder peak at 25.5° disappears and shifts toward a higher angle, approaching the intrinsic peak position of pure MWCNTs. This phenomenon indicates that high-content BiFeO_3_ induces the agglomeration of MWCNTs. The stress of MWCNTs inside the agglomerates is released, and the interplanar spacing recovers to the original state of pristine MWCNTs [21,22].

In addition, the position of the amorphous diffraction peaks of TPU in all composite samples remains unchanged with the variation in BiFeO_3_ content, revealing that the incorporation of BiFeO_3_ does not alter the average chain spacing of TPU molecular chains, and the amorphous structure of the TPU matrix remains stable [23,24]. Moreover, the intensity of TPU amorphous diffraction peaks decreases monotonously with the increasing BiFeO_3_ loading, which is mainly attributed to the dilution effect caused by the increased inorganic filler content [25,26,27].

### 4.2. Survey X-Ray Photoelectron Spectroscopy (XPS) Analysis of TPU/MWCNT/BiFeO_3_ Composites

**Figure 4:**
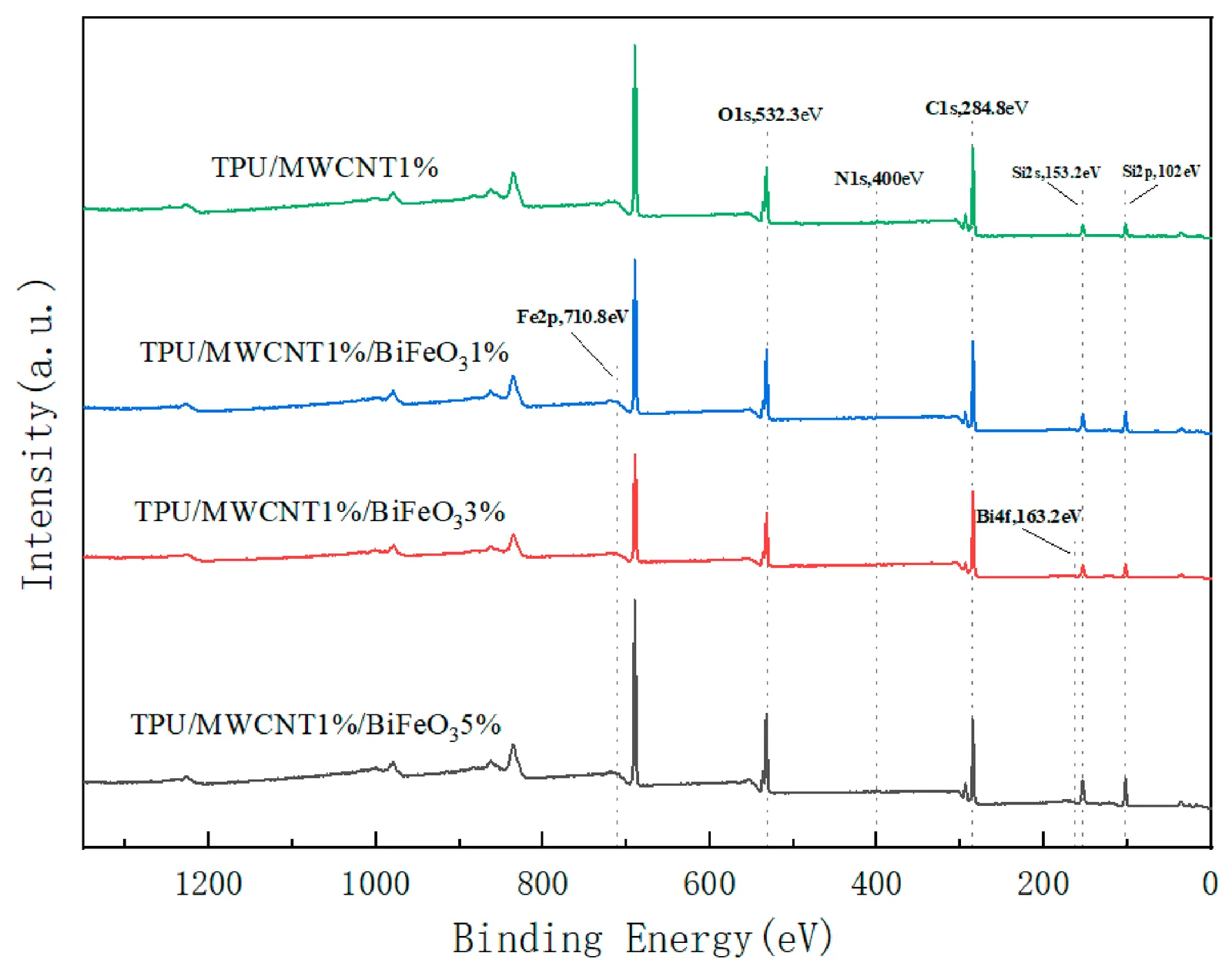
Full X-ray photoelectron spectroscopy (XPS) survey spectra of TPU/MWCNT/BiFeO_3_ composites.

The full X-ray photoelectron spectroscopy (XPS) survey spectra of TPU/MWCNT/BiFeO_3_ composites (Figure 4) illustrate the surface elemental composition of composites with different BiFeO_3_ contents. Characteristic peaks of C1s, O1s, N1s, Si2p, and Si2s were detected on the surface of all samples [28]. After the introduction of BiFeO_3_, weak Fe2p peaks appeared in the spectra, whereas the Bi4f signals were barely observable. For the TPU/MWCNT1% composite, the C1s, O1s, N1s, Si2s, and Si2p peaks are located at 284.8 eV, 532.3 eV, 400 eV, 153.2 eV, and 102 eV, respectively. The Fe2p peak of the TPU/MWCNT1%/BiFeO_3_1% composite is centered at 710.8 eV, while the Bi4f peak of the TPU/MWCNT1%/BiFeO_3_3% composite is located at 163.2 eV.

By comparing the XPS survey spectra of samples with different BiFeO_3_ contents, it can be observed that the height of the C1s peak shows no obvious variation with the increasing BiFeO_3_ loading, while the O1s peak height exhibits an overall upward trend. The intensities of the Si2p and Si2s peaks are also significantly enhanced, which is most prominent for the TPU/MWCNT1%/BiFeO_3_5% sample. The signals of N1s, Bi4f and Fe2p peaks are weak and hard to distinguish in the survey spectra.

Quantitative analysis based on XPS survey spectra (Table 3) further reveals the evolution of surface elemental composition. The Bi4f signals of all BiFeO_3_-containing samples are extremely weak (<0.02 at.%), and the atomic percentage of Fe2p ranges from 0.72% to 0.96%, without a monotonic increase with the rising BiFeO_3_ loading [29]. The total Si content (Si2s + Si2p) rises markedly from 16.59% for TPU/MWCNT1% to 32.89% for TPU/MWCNT1%/BiFeO_3_5% (16.7% for Si2s and 16.19% for Si2p). The oxygen content fluctuates between 20.86% and 23.72%, while the carbon content varies from 41.38% to 61.33%.

This phenomenon is closely related to the intrinsic characteristics of the BiFeO_3_ raw material, the residue of the JD-909A semi-permanent release agent used during compression molding, and residual DMF solvent. JD-909A is a commercial industrial release agent whose complete composition has not been disclosed by the manufacturer, and no peer-reviewed literature is currently available regarding its precise chemical makeup. Based on the industrial application background of similar semi-permanent release agents, it is inferred that the substantial amount of Si element detected in the samples originates from the silicone-based components of the release agent. This silicon species is considered an external contaminant on the sample surface rather than an intrinsic constituent of the TPU/MWCNT/BiFeO_3_ composite.

(1) Quantitative analysis of the Bi/Fe signals is presented as follows. As shown in Table 4, the surface atomic quantification results reveal that the Bi4f atomic percentage in all BiFeO_3_-containing samples is below 0.02 at.%, while the Fe2p atomic fraction falls within the range of 0.72–0.96 at.%, and the Fe content does not increase monotonically with increasing BiFeO_3_ filler loading. This observation can be rationalized by considering the filler particle size and the probing principle of XPS. The BiFeO_3_ powder used in the experiments is of −200 mesh size, with particle diameters smaller than 74 μm, which is far greater than the characteristic probing depth of XPS (approximately 10 nm). Consequently, the vast majority of BiFeO_3_ particles are encapsulated and buried beneath the TPU matrix polymer, so that XPS can only collect elemental information from within the topmost 10 nm of the sample surface, making it difficult to detect Bi elements from the particle interiors. Only a small fraction of particle fragments exposed on the matrix surface or iron-rich thin layers on the particle surfaces can contribute trace Fe signals. In addition, the residual release-agent layer covering the sample surface further shields the characteristic signals from the underlying inorganic particles, further suppressing the detected intensities of Bi and Fe. As a result, the effective Bi/Fe sources exposed at the material surface are quite limited, which explains why the Fe content does not increase with the total filler loading [30].
(2) Analysis of Si, O and C signals: The JD-909A release agent used during the compression molding process left significant residues on the sample surfaces, which served as the main source of Si element and also contributed substantially to the O and C signals [31]. The amount of release agent residue increased with rising BiFeO_3_ content (Si content rose from 16.59 at.% to 32.89 at.%), indicating that the higher BiFeO_3_ loading elevated the viscosity of the system, thereby leading to more release agent being transferred onto the sample surfaces. In addition, residual DMF solvent (boiling point 153 °C, drying temperature 140 °C) also contributed to a portion of the O and N signals [32]. The O1s content fluctuated in the range of 20.86–23.72 at.%, which is the result of the superposition of intrinsic oxygen from TPU, residual oxygen from DMF, and Si–O bond oxygen from the release agent.
(3) Analysis of N1s signal: The atomic percentage of N1s fluctuates between 0.49 at.% and 1.22 at.% without an obvious regular trend, which verifies the existence of trace nitrogen-containing residues (DMF) on the sample surface, yet their contribution to elemental signals is far lower than that of release agent residues.

### 4.3. FT-IR Spectroscopic Analysis of TPU/MWCNT/BiFeO_3_ Composites

**Figure 5:**
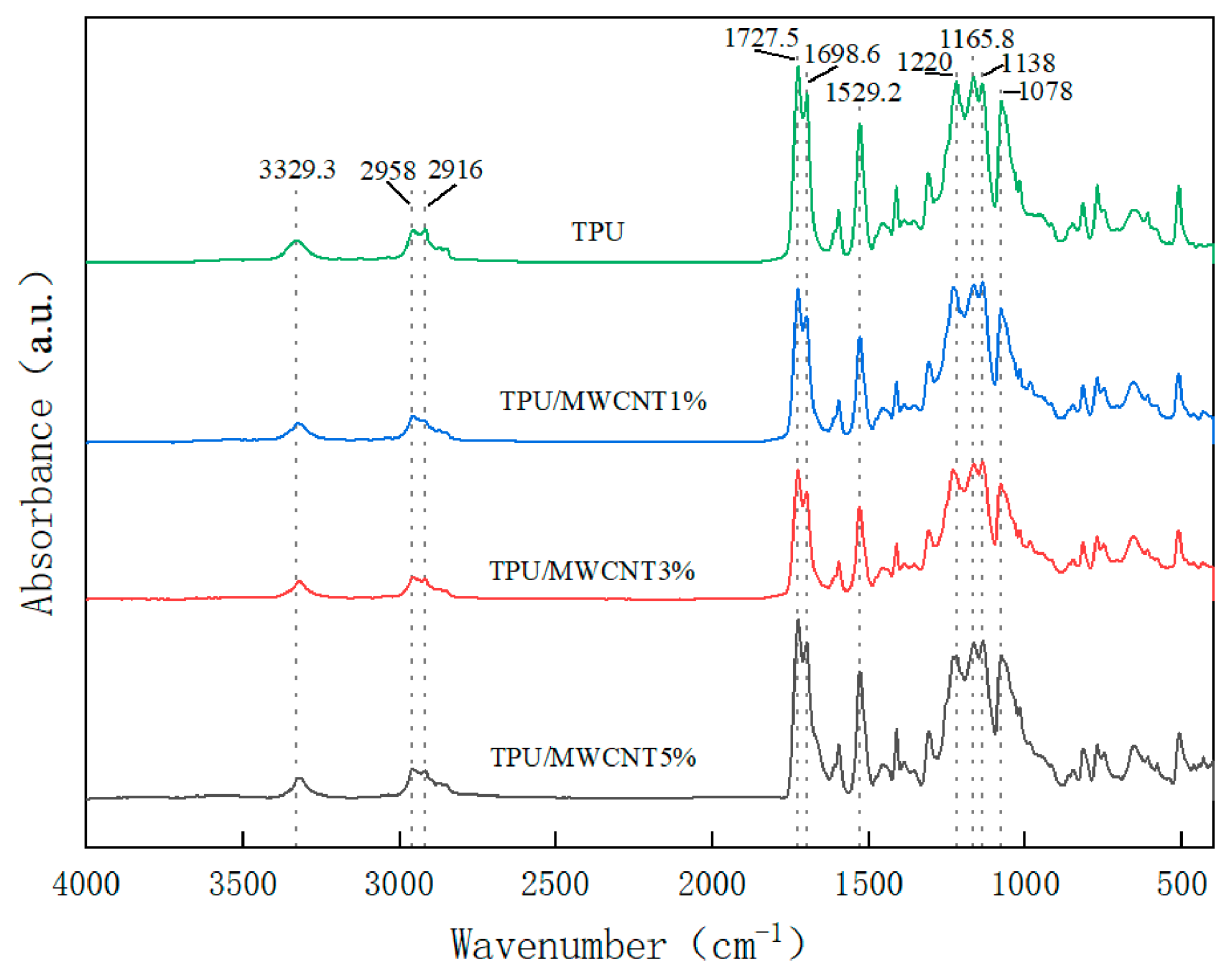
FT-IR spectra of TPU/MWCNT/BiFeO_3_ composites.

To investigate the effect of BiFeO_3_ incorporation on the microstructure of TPU/MWCNT/BiFeO_3_ composites, Fourier-transform infrared (FT-IR) spectroscopy analysis was performed on the TPU/MWCNT/BiFeO_3_ composites (Figure 5).

By comparing the Fourier-transform infrared spectra of TPU/MWCNT1% and TPU/MWCNT/BiFeO_3_ composites, no characteristic absorption peaks assigned to BiFeO_3_ (such as the Fe–O stretching vibration peak at approximately 550 cm^−1^) were detected in the spectra of all composites. This phenomenon can be attributed to the low loading content of BiFeO_3_ (≤5 wt%) and its micron-sized particles (−200 mesh), whose infrared signals were shielded by the strong absorption of the TPU matrix [33].

When the BiFeO_3_ content was 1 wt% and 5 wt%, the peak intensities of the carbonyl peaks of hard segments (1727.5 cm^−1^, 1700.6 cm^−1^), amide II band (1532.2 cm^−1^), and C–O–C ether bond peaks of soft segments (1230.4 cm^−1^, 1165.3 cm^−1^, 1136.9 cm^−1^, 1078.5 cm^−1^) all exhibited an upward trend. This is ascribed to two factors: the uniform dispersion of low-dose BiFeO_3_ strengthens interfacial interactions, and the overall confinement effect on molecular chains induced by the network structure formed by BiFeO_3_ at high loadings. Nevertheless, when the BiFeO_3_ loading reached 3 wt%, the intensities of all the above-mentioned peaks declined back to levels close to those of the TPU/MWCNT1% reference sample. This observation implies that 3 wt% may be the critical concentration at which BiFeO_3_ undergoes a transition from uniform dispersion to network formation within the matrix. Near this critical point, the original interfacial interactions are partially disrupted, while a completely new filler network has not yet been established, resulting in a temporary weakening of the confinement effect on the molecular chains of hard and soft segments.

Internal differentiation occurred for the C–H stretching vibration peaks attributed to soft segments at a BiFeO_3_ loading of 3 wt%: the intensity of the asymmetric C–H stretching peak (2959.7 cm^−1^) slightly decreased, while the intensity of the symmetric C–H stretching peak (2915.1 cm^−1^) marginally increased. Such differentiation further verifies the unique state at 3 wt%—the overall orderliness of soft segments declines (reflected by the weakened asymmetric stretching peak), yet the local mobility of molecular chains rises (reflected by the intensified symmetric stretching peak). This serves as a typical characteristic of the structural transition point [34,35].

The N–H stretching vibration peak of hard segments (3323.8 cm^−1^) slightly increased at all BiFeO_3_ loadings, demonstrating that interfacial hydrogen bonding interactions between BiFeO_3_ and hard segments persistently exist, yet such interactions are “masked” by the variation in filler dispersion state at the loading of 3 wt%.

### 4.4. Effect of BiFeO_3_ Content on Thermal Stability of TPU/MWCNT/BiFeO_3_ Composites

**Figure 6:**
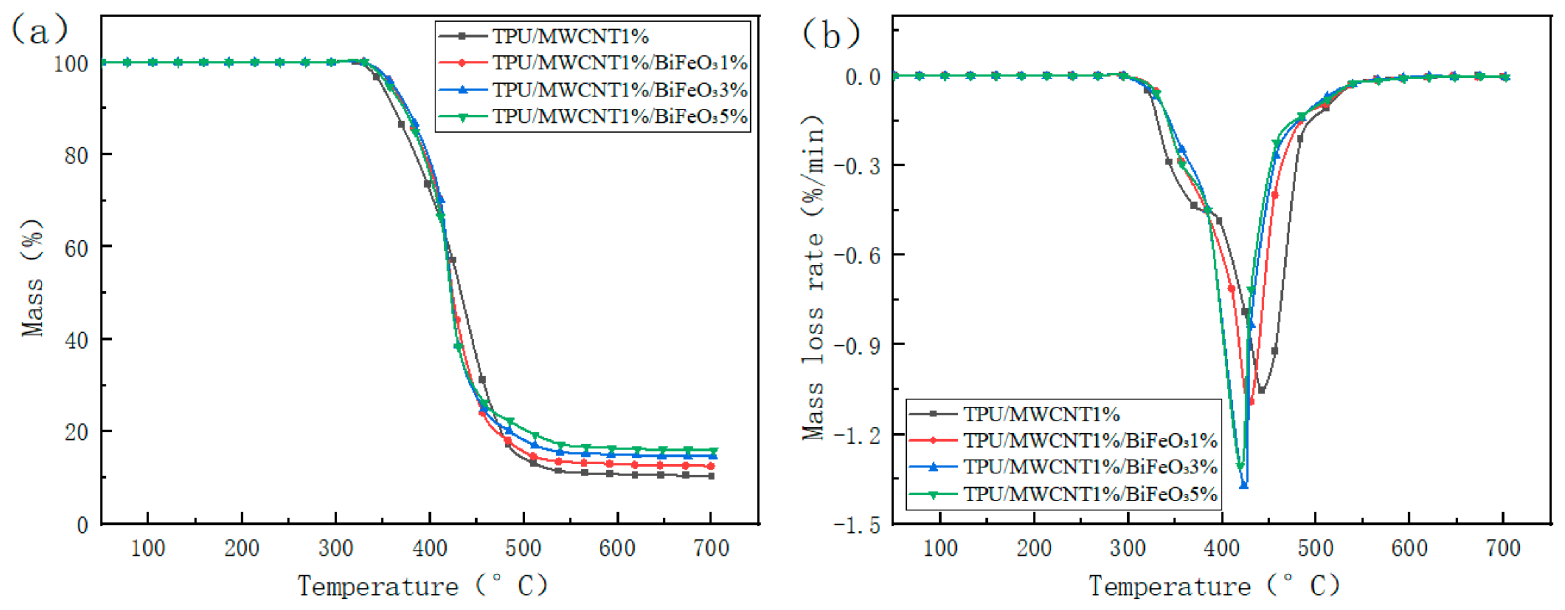
TPU/MWCNT/BiFeO_3_ composites: (**a**) Thermogravimetric curves; (**b**) Derivative thermogravimetric (DTG) curves.

Figure 6 displays the thermogravimetric analysis (TGA) and corresponding derivative thermogravimetry (DTG) curves of TPU/MWCNT/BiFeO_3_ composites. Combined with the data listed in Table 5, upon the introduction of BiFeO_3_, the composites exhibit an apparently contradictory thermal degradation behavior. The initial decomposition temperature (T5%) only slightly increases for samples filled with 1 wt% and 3 wt% BiFeO_3_, while the T5% value of the 5 wt% BiFeO_3_ sample is marginally lower than that of the TPU/MWCNT1% reference sample. As the BiFeO_3_ loading increases, the temperature at the maximum decomposition rate continuously decreases from 441.90 °C to 419.17 °C, accompanied by an increase in the absolute value of the maximum mass-loss rate. Meanwhile, the DTG curves transform from a two-stage decomposition profile into a single-peak pattern [36]. These observations indicate that BiFeO_3_ catalytically accelerates the main decomposition process of the TPU matrix, triggers preferential cleavage of hard-segment domains, and shifts the major degradation window toward lower temperatures. In the meantime, the char residue of the composites rises progressively from 10.42% to 16.01% with increasing BiFeO_3_ content. This phenomenon is mainly attributed to the outstanding high-temperature thermal stability of BiFeO_3_ itself and its char-promoting capacity, which facilitates the formation of a denser protective carbon layer [37,38].

With the increase in BiFeO_3_ loading, the temperature at the maximum decomposition rate continuously decreases from 441.90 °C to 419.17 °C, while the absolute value of the maximum mass loss rate rises from 1.054%/min to 1.372%/min. This indicates that the incorporation of BiFeO_3_ accelerates the reaction process in the main decomposition stage, allowing the material to reach its decomposition peak at a lower temperature. This observation is highly consistent with the morphological evolution of DTG curves. The DTG curve of TPU/MWCNT1% exhibits a typical two-stage decomposition feature with a shoulder peak on the left side of the main peak. After the addition of BiFeO_3_, the shoulder peak almost disappears, and the curve transforms into a nearly single peak. It can be inferred that BiFeO_3_ preferentially catalyzes or interferes with the decomposition of TPU hard segments, narrowing or advancing their decomposition temperature range to overlap with that of soft segments, thereby concentrating the thermal decomposition reaction [39].

The characteristic peaks of the gaseous pyrolysis products were assigned and analyzed based on the three-dimensional TGA-FTIR infrared spectra. The main gaseous products detected during thermal decomposition of the TPU-based composites and their corresponding characteristic wavenumbers are as follows: 3400–4000 cm^−1^ is attributed to the O–H stretching vibration of water vapor (H_2_O); 2800–3000 cm^−1^ corresponds to the C–H stretching vibrations of aliphatic hydrocarbons (–CH_2_–, –CH_3_), derived from the pyrolysis of the polyol soft-segment chains of TPU; 2360 cm^−1^ and 667 cm^−1^ are the characteristic absorption peaks of carbon dioxide (CO_2_), mainly originating from the cleavage of urethane hard-segment bonds; 1700–1760 cm^−1^ is assigned to the carbonyl C=O stretching vibration, corresponding to small pyrolysis molecules such as aldehydes, ketones, and esters; and 1500–1540 cm^−1^ is attributed to the NH-related vibration signals, arising from nitrogen-containing small molecules (amines, isocyanate fragments) produced by the decomposition of the polyurethane hard segments. No new inorganic gaseous decomposition products were observed in this system, indicating that BiFeO_3_ is thermally stable within the experimental pyrolysis temperature range and does not undergo significant gas-phase decomposition.

**Figure 7:**
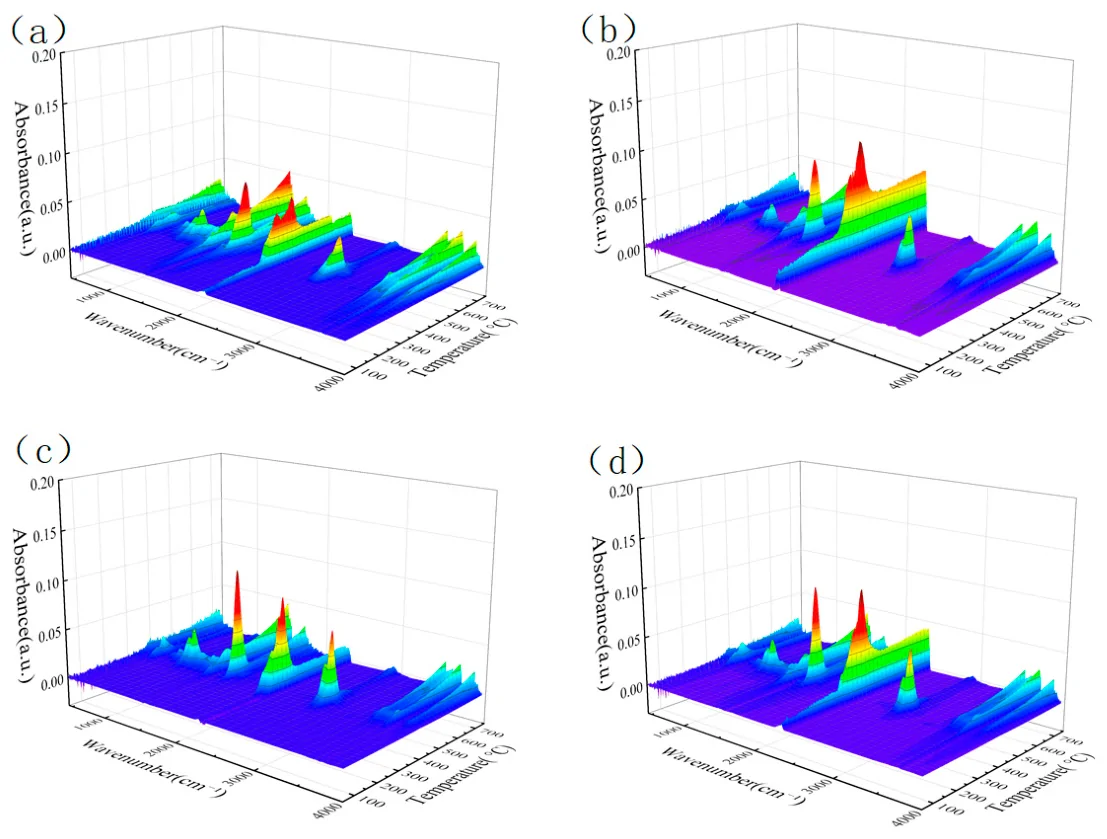
FTIR spectra of pyrolysis products from TPU/MWCNT/BiFeO_3_ composites: (**a**) TPU/MWCNT1%; (**b**) TPU/MWCNT1%/BiFeO_3_1%; (**c**) TPU/MWCNT1%/BiFeO_3_3%; (**d**) TPU/MWCNT1%/BiFeO_3_5%.

TGA-FTIR coupled characterization was carried out for further investigation. As displayed in Figure 7, all BiFeO_3_-containing samples present stronger peak intensities in their three-dimensional FTIR spectra compared with TPU/MWCNT1%, demonstrating that the incorporation of BiFeO_3_ accelerates the release of pyrolysis products or alters the composition of gaseous degradation products. Although the TPU/MWCNT1%/BiFeO_3_3% sample exhibits higher peak intensity than TPU/MWCNT1%, its absorbance values in the high-temperature region are lower instead. This phenomenon can be explained as follows: BiFeO_3_ with a loading of 3% catalyzes the rapid premature emission of partial pyrolysis products at the initial decomposition stage, which reduces the amount of gas available for escape at later temperatures. Alternatively, BiFeO_3_ may facilitate more pyrolytic fragments to participate in char-forming reactions and retain them in the condensed phase, thus leading to weaker gas emission signals under high-temperature conditions.

### 4.5. Fracture Morphology of TPU/MWCNT/BiFeO_3_ Composites

**Figure 8:**
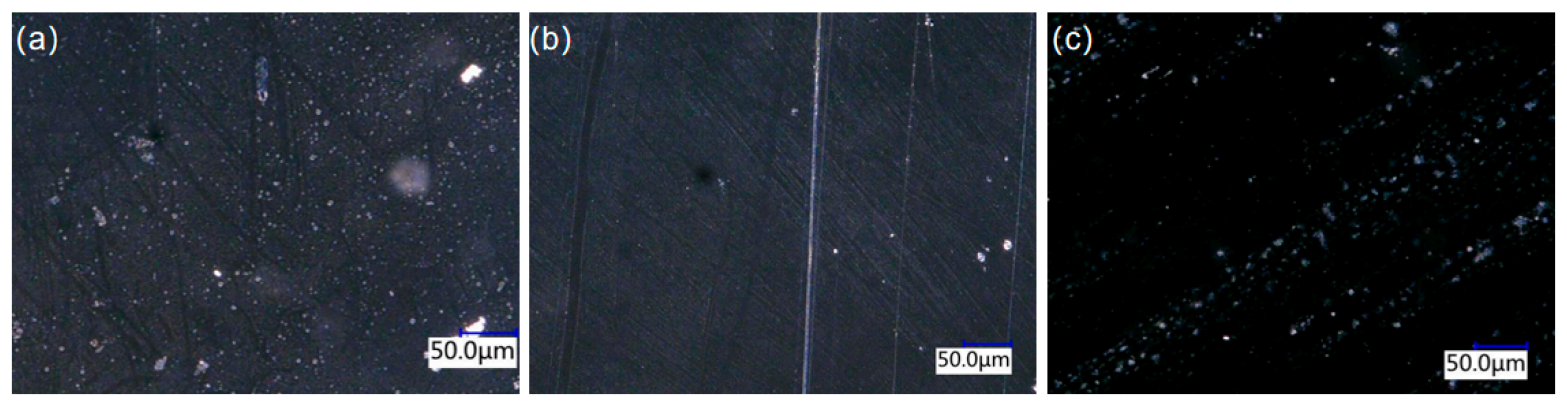
Super-depth-of-field microscope images of TPU/MWCNT composites (500× Scale bar: 50 μm): (**a**) TPU/MWCNT1%; (**b**) TPU/MWCNT3%; (**c**) TPU/MWCNT5%.

To preliminarily observe the dispersion state of BiFeO_3_ particles within the TPU/MWCNT matrix, the fracture surfaces of TPU/MWCNT/BiFeO_3_ composites were characterized using a super-depth-of-field microscope. Figure 8 presents the fracture morphologies of samples with various BiFeO_3_ loadings at a magnification of 500×.

**Figure 9:**
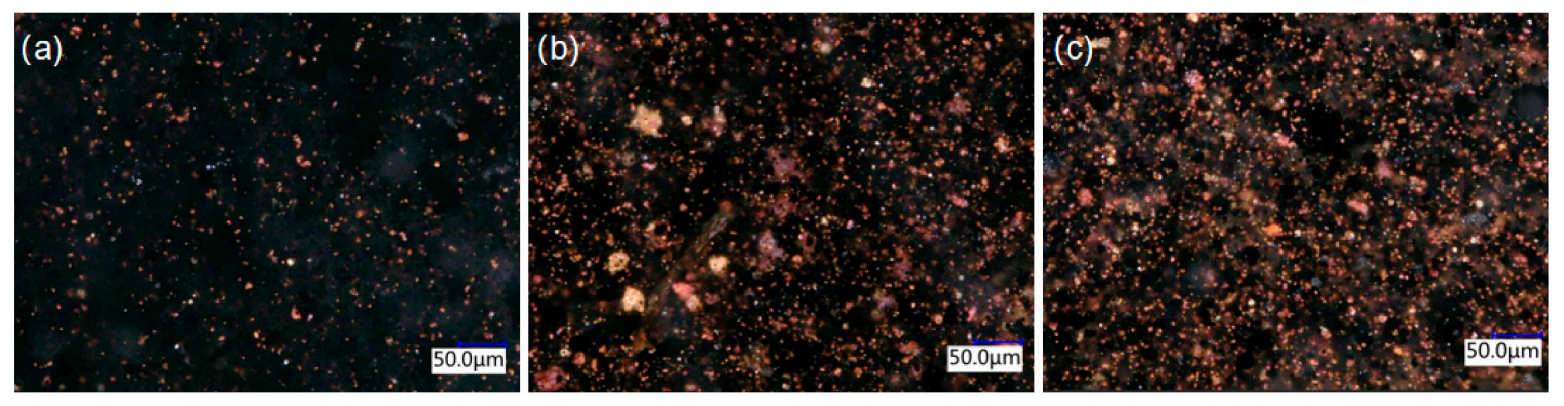
Super-depth-of-field Microscope Images of TPU/MWCNT/BiFeO_3_ Composites (500× Scale bar: 50 μm): (**a**) TPU/MWCNT1%/BiFeO_3_1%; (**b**) TPU/MWCNT1%/BiFeO_3_3%; (**c**) TPU/MWCNT1%/BiFeO_3_5%.

As can be seen from Figure 9, unlike the uniform dark background observed for the TPU/MWCNT sample (Figure 8), numerous scattered bright spots or light-colored particles emerge in the micrographs after the incorporation of BiFeO_3_. These bright spots correspond to pristine BiFeO_3_ particles and their agglomerates. As the BiFeO_3_ loading increases, the distribution density of bright spots within the field of view rises remarkably, indicating a greater number of observable BiFeO_3_ particles per unit area. Nevertheless, it can also be found that more large-sized, high-brightness agglomerates appear when the BiFeO_3_ loading reaches 3% and 5%. In particular, obvious particle agglomeration occurs in local regions of the 5% BiFeO_3_ sample. This reveals that BiFeO_3_ at high loadings tends to form agglomerates in the TPU/MWCNT matrix, leading to deteriorated dispersion uniformity.

This phenomenon can be attributed to the limited compatibility between inorganic ceramic BiFeO_3_ particles and the organic TPU matrix. Meanwhile, the van der Waals forces among particles strengthen with the rising particle concentration, which intensifies the tendency of agglomeration.

High-magnification super-depth-of-field microscopic observations reflect the dispersion status of BiFeO_3_ nanoparticles in the TPU/MWCNT matrix. It can be observed that numerous bright spots corresponding to BiFeO_3_ particles are dispersed in the matrix. The number of bright spots increases with BiFeO_3_ loading. Nevertheless, obvious local agglomeration occurs at higher filler content (3 wt% and 5 wt%), especially for the 5 wt% sample. This is mainly attributed to the poor compatibility between inorganic BiFeO_3_ ceramic particles and organic TPU matrix. Meanwhile, enhanced van der Waals forces at high particle loading further aggravate particle aggregation. Although SEM-EDS mapping was not performed in the present study, super-depth-of-field microscopic images still provide valid evidence to evaluate the dispersion and agglomeration of BiFeO_3_. Further elemental distribution characterization will be carried out in future research to quantitatively assess the agglomeration degree of BiFeO_3_ in TPU/MWCNT composites.

### 4.6. Effect of BiFeO_3_ Content on Tensile Properties of TPU/MWCNT/BiFeO_3_ Composites

**Figure 10:**
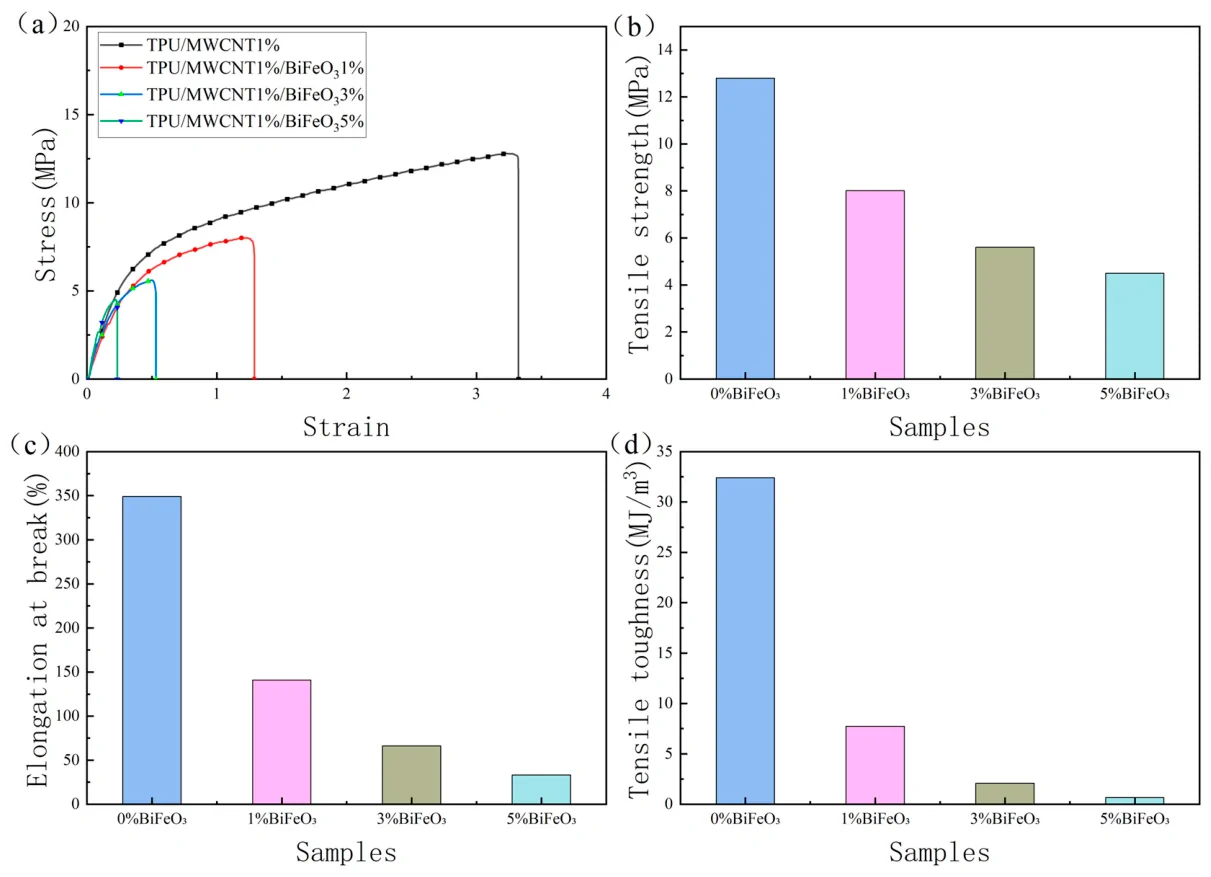
Mechanical properties of TPU/MWCNT/BiFeO_3_ composites: (**a**) Stress–strain curves; (**b**) Histogram of tensile strength; (**c**) Histogram of elongation at break; (**d**) Histogram of tensile toughness. (Note: In subgraphs (**b**–**d**), 0% BiFeO_3_ corresponds to TPU/MWCNT1%, 1% BiFeO_3_ corresponds to TPU/MWCNT1%/BiFeO_3_1%, 3% BiFeO_3_ corresponds to TPU/MWCNT1%/BiFeO_3_3%, and 5% BiFeO_3_ corresponds to TPU/MWCNT1%/BiFeO_3_5%).

Tensile tests were carried out on TPU/MWCNT/BiFeO_3_ composites with different BiFeO_3_ loadings (Figure 10). It can be observed from the stress–strain curves in Figure 10a that all samples exhibit the characteristics of filled elastomers with strain-softening behavior. The slope of each curve continuously decreases from the onset of stretching until fracture, without an obvious strain hardening stage.

With a fixed MWCNT loading of 1 wt%, BiFeO_3_ was incorporated into the matrix, and the tensile properties of the composites continuously deteriorated as the BiFeO_3_ content increased. As listed in Table 6, the tensile strength of the reference TPU/MWCNT1% sample is 12.80 MPa. After adding 1% BiFeO_3_, the tensile strength drops to 8.01 MPa with a reduction of 37.4%, followed by 5.60 MPa at 3% BiFeO_3_ (a 56.3% decrease), and only 4.49 MPa at 5% BiFeO_3_ (a 64.9% reduction). The elongation at break decreases from 349.10% for the reference sample to 140.79% (1% BiFeO_3_), 66.20% (3% BiFeO_3_) and 33.27% (5% BiFeO_3_), and the elongation of the 5% BiFeO_3_ sample merely accounts for 9.53% of the reference value. Tensile toughness suffers an even sharper decline from 32.40 MJ/m^3^ to 7.75 MJ/m^3^ (1% BiFeO_3_), 2.09 MJ/m^3^ (3% BiFeO_3_) and 0.67 MJ/m^3^ (5% BiFeO_3_). The toughness of the 5% BiFeO_3_ sample is almost completely lost, reaching only 2.1% of the reference sample.

The continuous deterioration of mechanical performance with increasing BiFeO_3_ loading can be attributed to the following reasons: (1) The incorporation of BiFeO_3_ particles introduces dilution and disruption effects on the existing MWCNT conductive network [40]. XRD results confirm that high BiFeO_3_ content induces agglomeration of MWCNTs. The micron-sized BiFeO_3_ particles are dispersed in the interstitial spaces among the interconnected MWCNTs, physically blocking the effective contact sites between carbon nanotubes and thereby destroying the physical crosslinking network constructed by MWCNTs within the TPU matrix. In addition to providing conductive pathways, MWCNTs also act as physical crosslinking points that constrain TPU molecular chains and bear part of the external load. When the continuity of this network is interfered with by the inorganic particles, the load cannot be effectively transferred during matrix deformation, directly resulting in a decrease in tensile strength and toughness. (2) The intrinsic interfacial compatibility between the inorganic BiFeO_3_ ceramic filler and the organic TPU matrix is poor. BiFeO_3_ is a perovskite-structured inorganic oxide with a highly polar surface, whereas the TPU molecular chains contain polar urethane groups; nevertheless, only a limited number of hydrogen-bonding interactions can be established between them, and strong chemical bonding is absent. During tensile deformation, debonding tends to occur preferentially at the interface between BiFeO_3_ particles and the TPU matrix, generating microvoids at the interfacial regions, which subsequently evolve into crack initiation sites. Under sustained external loading, these microcracks rapidly propagate and coalesce, leading to premature fracture of the material, which is macroscopically reflected by a substantial decrease in elongation at break. FT-IR results indicate that BiFeO_3_ can form certain hydrogen bonds with the hard segments of TPU; however, this interfacial hydrogen-bonding interaction is rather limited and insufficient to compensate for the interfacial defects arising from the intrinsic incompatibility between the inorganic and organic phases. (3) Filler agglomeration further amplifies the stress concentration effect. Ultra-depth-of-field microscopy observations of the fracture surface morphology reveal that when the BiFeO_3_ content is increased to 3 wt% and 5 wt%, distinct BiFeO_3_ agglomerates appear within the matrix. Within the agglomerated regions, there is almost no TPU matrix infiltration between the particles, and a large number of inherent micro-defects exist internally. Upon tensile loading, stress becomes highly concentrated at the boundaries of the agglomerated particles, and cracks preferentially initiate at the agglomerate sites and propagate rapidly. The higher the filler loading, the greater the probability of agglomeration, the larger the defect size, and the poorer the ultimate deformability that the material can withstand.

The synergistic effect of these three factors comprehensively degrades the strength, ductility and toughness of the composites. Notably, the sample filled with 5 wt% BiFeO_3_ nearly loses typical elastomeric characteristics and evolves toward brittle material behavior.

It should be noted that the improvement of electromagnetic shielding effectiveness is accompanied by obvious deterioration of ductility when BiFeO_3_ loading reaches 3 wt%. Although the introduction of BiFeO_3_ contributes to enhanced interfacial polarization and magnetic loss for better EMI shielding performance, the rigid inorganic BiFeO_3_ particles exert negative effects on the toughness of TPU/MWCNT composites. On the one hand, excessive BiFeO_3_ nanoparticles tend to form agglomerates within TPU matrix, which act as stress-concentration sites under external tension and induce early crack initiation and propagation. On the other hand, rigid BiFeO_3_ particles restrict the mobility of flexible TPU molecular chains. In addition, the poor interfacial compatibility between inorganic BiFeO_3_ and organic TPU weakens interfacial stress transfer, further resulting in a sharp decline in elongation at break and toughness. Such “performance trade-off” is a common challenge for magnetic-filled TPU-based electromagnetic shielding composites. In follow-up work, surface modification of BiFeO_3_ filler will be adopted to ameliorate interfacial compatibility, so as to achieve balanced electromagnetic shielding and mechanical properties.

### 4.7. Effect of BiFeO_3_ Content on Electromagnetic Interference Shielding Performance of TPU/MWCNT/BiFeO_3_ Composites

**Figure 11:**
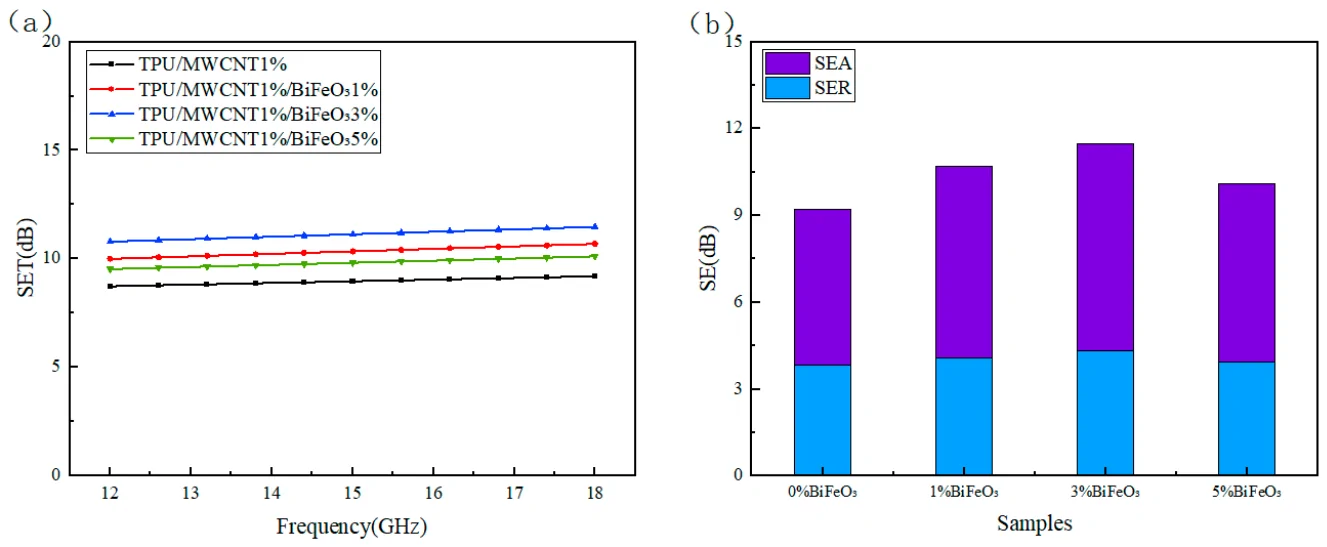
Electromagnetic interference shielding characteristics of TPU/MWCNT/BiFeO_3_ composites: (**a**) Total shielding effectiveness (SET); (**b**) Comparison of SEA and SER at 18 GHz. (Note: The abbreviations of samples are consistent with those in Figure 10).

Figure 11 presents the electromagnetic interference shielding effectiveness (SE) of TPU/MWCNT/BiFeO_3_ composites with different BiFeO_3_ loadings in the frequency range of 12.4–18 GHz. As shown in Figure 11a, the maximum SE value of the composites rises first and then declines with increasing BiFeO_3_ content. Without BiFeO_3_ (i.e., TPU/MWCNT1%), the maximum SE is 9.20 dB. After incorporating 1 wt% BiFeO_3_, the maximum SE increases to 10.69 dB. When the BiFeO_3_ loading rises to 3 wt%, the maximum SE reaches a peak value of 11.47 dB, approximately 24.7% higher than that of the TPU/MWCNT1% sample. Nevertheless, further increasing the BiFeO_3_ loading to 5 wt% causes the maximum SE to drop to 10.11 dB.

Figure 11b compares the values of absorption shielding efficiency (SEA) and reflection shielding efficiency (SER) for all samples at 18 GHz. According to the histogram data, both SEA and SER show an upward-then-downward trend with increasing BiFeO_3_ loading, and SEA exhibits a more prominent variation range. For the sample without BiFeO_3_ filler, SEA is 5.35 dB and SER is 3.84 dB. At a BiFeO_3_ loading of 1 wt%, SEA rises to 6.61 dB and SER increases to 4.08 dB. When the loading reaches 3 wt%, SEA and SER reach their maximum values of 7.16 dB and 4.32 dB, respectively. Further raising the BiFeO_3_ loading to 5 wt% causes SEA and SER to decline to 6.16 dB and 3.95 dB, respectively.

This reveals that the incorporation of BiFeO_3_ primarily improves the wave absorption loss capacity of the composites. Such improvement originates from interfacial polarization, dipole polarization and multiple scattering effects induced by ferroelectric BiFeO_3_, which accelerate the dissipation of electromagnetic waves inside the material. Nevertheless, excessive BiFeO_3_ particles tend to agglomerate in the matrix, breaking the conductive network constructed by MWCNTs and worsening impedance matching, which ultimately reduces the electromagnetic interference shielding effectiveness [41].

### 4.8. Analysis of Electromagnetic Shielding Mechanism of TPU/MWCNT/BiFeO_3_ Composite Materials

**Figure 12:**
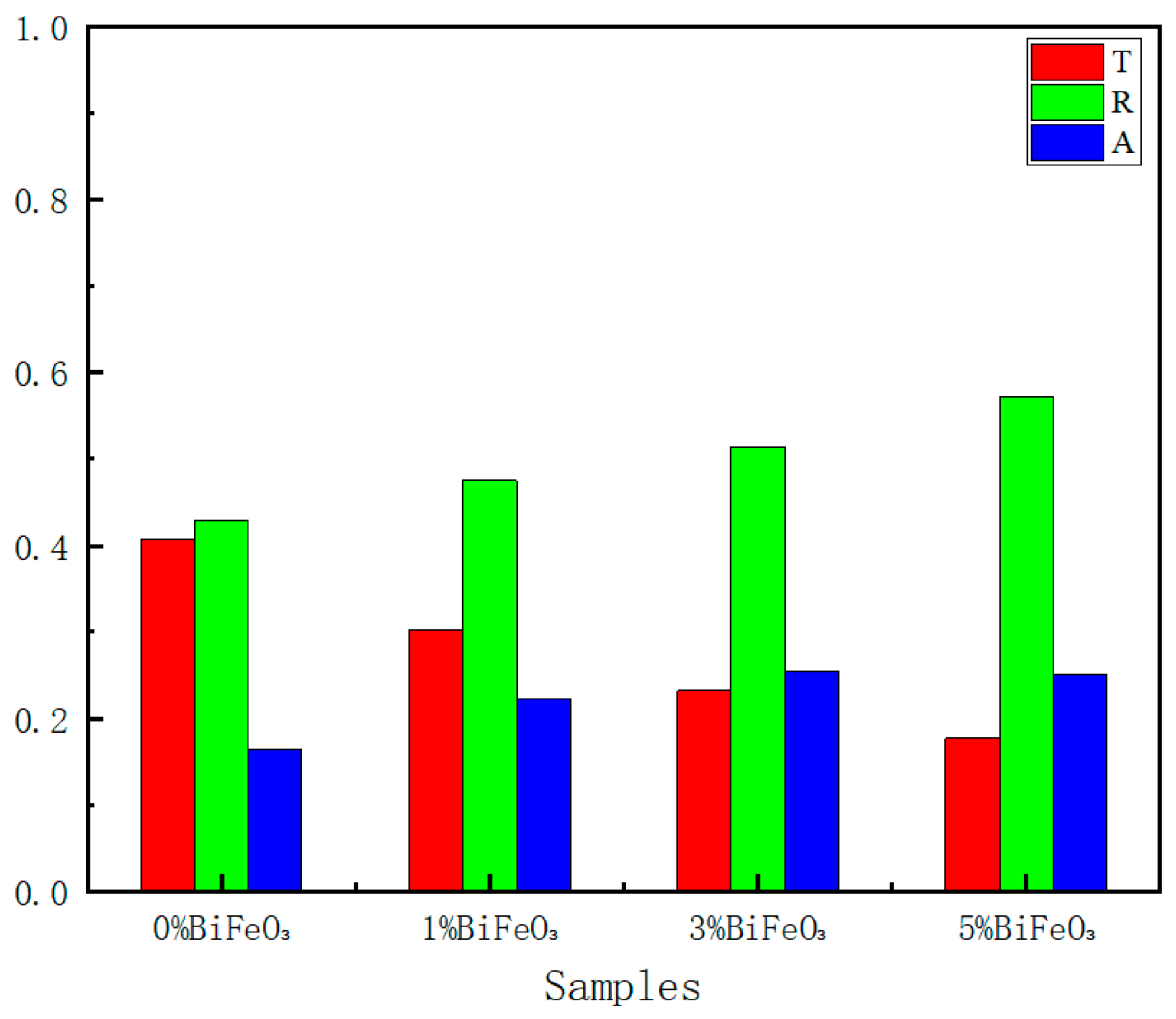
Transmission coefficient (T), reflection coefficient (R) and absorption coefficient (A) of TPU/MWCNT/BiFeO_3_ composites at 18 GHz. (Note: The abbreviations of samples are consistent with those in Figure 10).

To deeply investigate the effect of BiFeO_3_ loading on the electromagnetic shielding mechanism of TPU/MWCNT/BiFeO_3_ composites, Figure 12 presents the transmission coefficient (T), reflection coefficient (R) and absorption coefficient (A) of TPU/MWCNT/BiFeO_3_ composites at 18 GHz. It can be observed from the plotted data that the transmission coefficient T of the composites decreases continuously with the rising BiFeO_3_ content. The transmission coefficient T reaches 0.41 for the sample without BiFeO_3_ (namely TPU/MWCNT with 1 wt% MWCNT). When the BiFeO_3_ loading is 1 wt%, 3 wt% and 5 wt%, the transmission coefficient T declines to 0.30, 0.23 and 0.18, respectively. This reveals that the incorporation of BiFeO_3_ effectively blocks the penetration of electromagnetic waves and progressively improves the shielding performance.

The reflection coefficient R shows a monotonically increasing trend with the increase in BiFeO_3_ content, rising from 0.43 at 0 wt% BiFeO_3_ to 0.57 at 5 wt% BiFeO_3_. This is attributed to the introduction of BiFeO_3_ ferroelectric filler, which further aggravates the impedance mismatch between the composite and air, leading to more electromagnetic waves being reflected at the material surface. In contrast, the absorption coefficient A first rises and then slightly declines as BiFeO_3_ loading increases. The absorption coefficient A increases from 0.16 to 0.22 at 1 wt% BiFeO_3_, and reaches a maximum value of 0.25 at a BiFeO_3_ loading of 3 wt% [42].

The electromagnetic wave attenuation in this system originates from the synergistic effect of three mechanisms: conductive loss from MWCNTs, dielectric loss from BiFeO_3_, and weak antiferromagnetic magnetic loss. MWCNTs interconnect within the TPU matrix to form a continuous conductive network, where the migration of free electrons under an alternating electromagnetic field generates ohmic loss, constituting the fundamental pathway for electromagnetic wave attenuation. BiFeO_3_, as a room-temperature antiferromagnetic–ferroelectric multiferroic material, possesses theoretical magnetic response potential; however, due to the helical spin-modulated crystal structure of BiFeO_3_, the intrinsic magnetic loss in the 12.4–18 GHz Ku band is relatively weak. Consequently, magnetic loss plays only a minor auxiliary role in this system, and dielectric loss remains the dominant dissipation mechanism within the composite. The dielectric loss primarily arises from three contributions: (i) dipole polarization induced by oxygen vacancies and lattice defects within the BiFeO_3_ particles; (ii) interfacial polarization generated at multiphase heterogeneous interfaces, including TPU–BiFeO_3_, TPU–MWCNT, and MWCNT–BiFeO_3_ interfaces, where numerous interfacial polarization centers can dissipate the electromagnetic wave energy entering the material [43]; (iii) multiple scattering and reflection of electromagnetic waves between filler particles, which prolongs the propagation path of the electromagnetic waves and further consumes electromagnetic energy.

In summary, the electromagnetic shielding performance of TPU/MWCNT/BiFeO_3_ composites originates from the synergistic effect of reflection loss and absorption loss. Notably, absorption loss is greatly boosted after BiFeO_3_ doping and achieves the optimal shielding effect at a BiFeO_3_ loading of 3 wt%.

**Figure 13:**
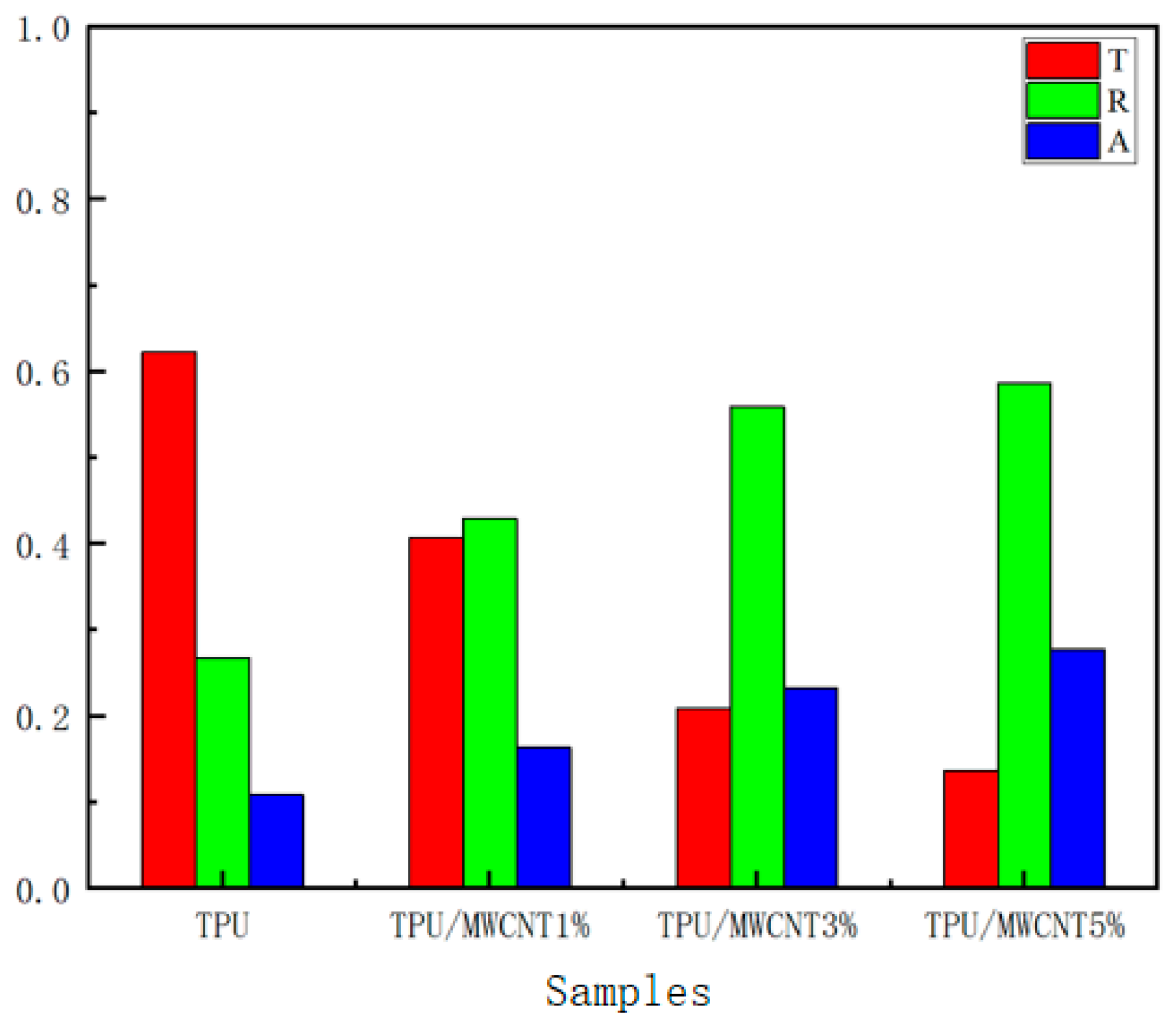
Transmission coefficient (T), reflection coefficient (R), and absorption coefficient (A) of TPU/MWCNT composites at 18 GHz.

As shown in Figure 13. The transmission coefficient T refers to the fraction of electromagnetic wave energy transmitted through the material, whereas the reflection coefficient R and absorption coefficient A represent the energy fractions of electromagnetic waves reflected and dissipated via absorption by the material, respectively. It can be seen from the data that neat TPU delivers a high transmission coefficient T of 0.62, a reflection coefficient R of 0.27 and an absorption coefficient A of merely 0.11. This demonstrates that neat TPU has almost no EMI shielding performance, and most electromagnetic waves penetrate directly through the material.

After incorporating MWCNT, the transmission coefficient T of the composite decreases significantly. At an MWCNT loading of 1 wt%, the transmission coefficient T drops to 0.41, and it further decreases to 0.21 and 0.13 as the loading increases to 3 wt% and 5 wt%, respectively. This indicates that the incorporation of MWCNT effectively hinders the transmission of electromagnetic waves. Meanwhile, both the reflection coefficient R and absorption coefficient A increase with rising MWCNT content. The reflection coefficient R increases from 0.41 for TPU/MWCNT1% to 0.59 for TPU/MWCNT5%, which is attributed to the enhanced impedance mismatch between the MWCNT-constructed conductive network and air, causing more electromagnetic waves to be reflected at the material surface. The absorption coefficient A rises from 0.11 for neat TPU to 0.28 for TPU/MWCNT5%. At the MWCNT loading of 5 wt%, the absorption coefficient A reaches 0.28, accompanied by a reflection coefficient R of 0.59 and a transmission coefficient T of merely 0.13. The continuous growth of the absorption coefficient A demonstrates that electromagnetic waves entering the interior of the material are efficiently dissipated. Such dissipation mainly originates from mechanisms including ohmic loss, multiple-reflection effect and interfacial polarization within the MWCNT-built conductive network. In summary, the EMI shielding performance of TPU/MWCNT composites stems from the combined effects of reflection loss and absorption loss. The introduction of MWCNT not only strengthens the surface reflection of electromagnetic waves, but more importantly, greatly improves the absorption capacity toward electromagnetic waves that penetrate into the material.

It is instructive to compare the present work with the previously reported TPU/MWCNT binary electromagnetic interference (EMI) shielding composites and polymer/BiFeO_3_-based electromagnetic functional materials. For instance, the iPP/TPU/MWCNT composites prepared by Li et al. required a high MWCNT loading of up to 10 wt% to achieve excellent shielding effectiveness, with the shielding mechanism relying predominantly on the conductive loss of carbon nanotubes and lacking a dielectric loss contribution [1]. In carbon-nanotube-filled polymer systems, a common issue is the deterioration of mechanical properties at high filler loadings. In contrast, the present work employs only 1 wt% MWCNT in combination with a small amount of BiFeO_3_ dielectric filler, establishing a “conductive–dielectric” synergistic loss mechanism that enhances shielding performance at a relatively low total filler content.

Existing studies on polymer/BiFeO_3_ systems have primarily focused on nanosized BiFeO_3_ fillers or on standalone microwave-absorbing materials, and it has been established that micron-sized BiFeO_3_ particles tend to agglomerate more readily in polymer matrices [44]. In this work, 200-mesh micron-sized BiFeO_3_ particles were employed to systematically investigate the microscopic behavior of such micron-fillers within the TPU/MWCNT conductive matrix. It was clearly observed that with increasing BiFeO_3_ content, filler agglomeration became progressively more severe. Meanwhile, this study reveals a significant shielding–mechanical property trade-off effect in this ternary system: the enhancement in shielding performance is accompanied by a substantial deterioration in material toughness, and the sample with 5 wt% BiFeO_3_ largely loses its elastomeric characteristics. This trade-off phenomenon has often been overlooked in similar composite shielding systems.

## 5. Conclusions

In this experiment, with the MWCNT loading fixed at 1 wt%, BiFeO_3_ particles with varying loadings (0 wt%, 1 wt%, 3 wt%, 5 wt%) were incorporated. Ternary TPU/MWCNT/BiFeO_3_ composites were successfully fabricated via solution blending followed by hot-pressing molding. The effects of BiFeO_3_ loading on the microstructure, thermal stability, mechanical properties and electromagnetic interference shielding effectiveness (EMI SE) of the composites were systematically investigated. Particular emphasis was placed on discussing the synergistic effect and electromagnetic wave attenuation law under the dual mechanisms of conductive loss and dielectric loss. The main conclusions are summarized as follows:

(1) Microstructural characterization revealed the following findings. XRD confirmed that BiFeO_3_ was successfully introduced into the TPU matrix, with the intensity of the characteristic diffraction peaks of BiFeO_3_ gradually increasing with rising filler loading. At high BiFeO_3_ contents, agglomeration of MWCNTs was induced, causing the characteristic peaks of MWCNT crystal planes to shift toward higher angles. XPS analysis showed that, owing to the particle size of BiFeO_3_ (<74 μm) being far larger than the probing depth of XPS (approximately 10 nm), together with the shielding effect of silicone-based residues from the JD-909A release agent on the sample surface, the Bi signal was extremely low (<0.02 at.%), and only trace amounts of Fe could be detected. FT-IR spectra indicated that BiFeO_3_ could form hydrogen-bonding interactions with the hard segments of TPU; a filler loading of 3 wt% was identified as the critical concentration marking the transition from uniform dispersion to a filler network, at which the constraining effect on the molecular chains of the soft and hard segments was temporarily weakened. Ultra-depth-of-field microscopy observations of the cross-sectional morphologies demonstrated that BiFeO_3_ agglomerated within the matrix, and the degree of agglomeration intensified with increasing filler content; large-sized agglomerates were clearly observable in the 5 wt% sample [45].
(2) Thermogravimetric analysis (TGA) was adopted to investigate the thermal stability of TPU/MWCNT/BiFeO_3_ composites. BiFeO_3_ exerts a catalytic char-forming effect on the TPU matrix. It accelerates the degradation in the main decomposition stage and shifts the temperature at the maximum decomposition rate toward lower temperatures. Meanwhile, it promotes the generation of condensed-phase char, increasing the char residue from 10.42% to 16.01%. The T5% values show only minor variations for the 1 wt% and 3 wt% BiFeO_3_ samples, whereas a decline in T5% is observed for the 5 wt% BiFeO_3_ sample. TGA-FTIR results verify that BiFeO_3_ accelerates the thermal pyrolysis. The reduction in gas-phase products in the high-temperature region for the 3 wt% sample indicates that more pyrolytic intermediates are converted into solid-phase char layers, while T_5_% decreased at a loading of 5 wt%, revealing that excessive BiFeO_3_ might trigger slight early-stage decomposition. The char residue rate continuously increased from 10.42% to 16.01% with rising BiFeO_3_ loading, which originated from the char-promoting effect of BiFeO_3_. Nevertheless, the temperature corresponding to the maximum decomposition rate declined steadily from 441.90 °C to 419.17 °C, and the DTG curves transformed from two-stage decomposition profiles into single peaks. This indicates that BiFeO_3_ accelerates the reaction rate of the main decomposition stage and preferentially catalyzes the decomposition of hard segments in TPU. TGA-FTIR characterization shows that the release intensity of pyrolysis products from BiFeO_3_-containing samples is generally higher than that of the BiFeO_3_-free system. Notably, the absorbance of the sample with 3 wt% BiFeO_3_ decreases significantly in the high-temperature region, demonstrating that BiFeO_3_ at this loading level can facilitate the participation of pyrolysis products in char-forming reactions and thereby suppress gas emission at high temperatures [46].
(3) The tensile properties of the TPU/MWCNT/BiFeO_3_ composites were systematically investigated. With a fixed MWCNT content of 1 wt%, the tensile strength, elongation at break, and tensile toughness continuously deteriorated with increasing BiFeO_3_ loading. Compared with the benchmark sample TPU/MWCNT1%, the TPU/MWCNT1%/BiFeO_3_5% composite exhibited a decrease in tensile strength from 12.80 MPa to 4.49 MPa, a reduction in elongation at break from 349.10% to 33.27%, and a decline in tensile toughness from 32.40 MJ/m^3^ to 0.67 MJ/m^3^. This phenomenon is attributed to the following mechanisms: the BiFeO_3_ particles disrupt the MWCNT physical crosslinking network, the weak interfacial bonding between the inorganic and organic phases leads to interfacial debonding, severe stress concentration is induced by filler agglomeration at high loadings, and the rigid particles restrict the mobility of the soft segment molecular chains of TPU. The 5 wt% specimen essentially lost its elastomeric characteristics. Consequently, there exists a trade-off effect between electromagnetic interference (EMI) shielding performance and mechanical properties; although the 3 wt% BiFeO_3_ formulation gave the optimal shielding performance, its mechanical properties had already deteriorated markedly. Future efforts may focus on surface modification of the filler to improve interfacial compatibility, thereby achieving a synergy between shielding effectiveness and mechanical performance.
(4) The influence of BiFeO_3_ loading on the electromagnetic interference shielding performance of TPU/MWCNT/BiFeO_3_ composites was investigated, and the corresponding electromagnetic shielding mechanism was analyzed:① The total shielding effectiveness (SE_t_) of the composites exhibited an upward-then-downward trend with the increase in BiFeO_3_ loading. At the frequency of 18 GHz, the maximum SE_t_ of TPU/MWCNT-1 wt%/BiFeO_3_-3 wt% reached 11.47 dB, approximately 24.7% higher than that of TPU/MWCNT-1 wt% (9.20 dB). Nevertheless, when BiFeO_3_ loading rose to 5 wt%, the maximum SE_t_ decreased to 10.11 dB. Both absorption loss (SEₐ) and reflection loss (SEᵣ) followed the identical variation tendency, and the fluctuation range of SEₐ was much more remarkable.② As BiFeO_3_ loading increased, the transmission coefficient (T) decreased continuously from 0.41 to 0.18, while the reflection coefficient (R) increased monotonically from 0.43 to 0.57. By contrast, the absorption coefficient (A) first increased and then slightly declined, peaking at 0.25 at 3 wt% BiFeO_3_. This reveals that the incorporation of BiFeO_3_ mainly strengthens the absorption loss capacity of the composites. The underlying mechanisms are as follows: BiFeO_3_ acts as a ferroelectric filler to generate interfacial polarization and dipole polarization effects; numerous multi-interfacial polarization centers are constructed among BiFeO_3_, MWCNT and TPU matrix, which facilitate the dissipation of electromagnetic waves inside the composite. However, severe agglomeration of excessive BiFeO_3_ (5 wt%) breaks the continuity of the MWCNT conductive network and deteriorates impedance matching, thereby reducing the shielding effectiveness.

Although the TPU/MWCNT/BiFeO_3_ composite realizes conductive-dielectric synergistic EMI shielding, limitations still exist, such as easy agglomeration of micro-BiFeO_3_ and weak magnetic-loss contribution in the Ku-band. Future work will focus on BiFeO_3_ nanocrystallization and surface modification to improve filler dispersion and exploit its magnetic-loss potential. This work provides experimental data and a theoretical reference for developing flexible TPU-based EMI shielding materials.

## Figures and Tables

**Table 1:** Experimental raw materials.

Raw Materials	Specifications and Models	Manufacturer
BiFeO_3_	99.9% (BiFeO_3_)-200mesh	Zhongnuo New Material Tech. Co., Ltd., Xuzhou, China
MWCNT	NC7000	Jiangsu Xianfeng Nano Material Tech. Co., Ltd., Nanjing, China
TPU	Polyester type, transparent white, hardness 89A	Dongguan Jinheng Plastic Co., Ltd., Dongguan, China
DMF	Specification: >99.9% (GC)	Shanghai Macklin Biochemical Co., Ltd., Shanghai, China
Semi-permanent release agent	JD-909A	Dongguan Jiadan Lubricating Oil Co., Ltd., Dongguan, China

**Table 2:** Experimental equipment.

Equipment Name	Equipment Model	Manufacturer
High-speed mixer	JB-260SH	Shanghai Yike Scientific Instrument Co., Ltd., Shanghai, China
Electronic balance	JA203	Changzhou Xingyun Equipment Co., Ltd., Changzhou, China
Vacuum drying oven	ZKXF	Shanghai Shuli Co., Ltd., Shanghai, China
Manual Vacuum Plate Vulcanizing Machine	CH-0206	Dongguan Chuanghong Instrument & Equipment Co., Ltd., Dongguan, China
Simultaneous thermal analyzer	STA8000	PerkinElmer Corporation, Shelton, CT, USA
Fourier Transform Infrared Spectrometer	SP3	PerkinElmer Corporation, Shelton, CT, USA
Super-depth-of-field microscope	VHX-5000	Keyence, Osaka, Japan
Vector Network Analyzer	Ceyear 3672D	Beijing Oriental Zhongke Integrated Technology Co., Ltd., Beijing, China
Universal Testing Machine	DNS100	Jinan Huaxing Test Equipment Co., Ltd., Jinan, China
X-ray diffractometer	Ultima IV	Rigaku Corporation, Tokyo, Japan
X-ray photoelectron spectrometer	Kratos AXIS SUPRA	Kratos Analytical Ltd., Manchester, UK

**Table 3:** Component proportions of TPU/MWCNT/BiFeO_3_ composites.

Samples	TPU (g)	Mass Fraction of MWCNT (wt%)	BiFeO_3_ (g)	Mass Fraction of BiFeO_3_ (wt%)
TPU/MWCNT 1%	45	1	0	0
TPU/MWCNT 1%/BiFeO_3_1%	45	1	0.45	1
TPU/MWCNT 1%/BiFeO_3_3%	45	1	1.35	3
TPU/MWCNT 1%/BiFeO_3_5%	45	1	2.25	5

**Table 4:** Relative atomic percentages (at.%) of surface elements for TPU/MWCNT/BiFeO_3_ composites.

Samples	C1s (at.%)	O1s (at.%)	N1s (at.%)	Si2s (at.%)	Si2p (at.%)	Bi4f (at.%)	Fe2p (at.%)
TPU/MWCNT1%	61.33	20.86	1.22	8.71	7.88	0	0
TPU/MWCNT1%/BiFeO_3_1%	50.01	22.84	0.49	12.83	12.87	0	0.96
TPU/MWCNT1%/BiFeO_3_3%	57.82	20.99	0.53	10.08	9.85	0.01	0.72
TPU/MWCNT1%/BiFeO_3_5%	41.38	23.72	1.13	16.7	16.19	0.01	0.86

**Table 5:** Thermal analysis parameters of TPU/MWCNT/BiFeO_3_ composites.

Samples	T_5_% (°C)	Maximum Mass Loss Rate (%/min)	Temperature at the Maximum Mass Loss Rate (°C)	Char Residue Yield (%)
TPU/MWCNT1%	348.74	−1.054	441.90	10.42%
TPU/MWCNT1%/BiFeO_3_1%	351.75	−1.090	430.36	12.52%
TPU/MWCNT1%/BiFeO_3_3%	352.63	−1.372	422.92	14.70%
TPU/MWCNT1%/BiFeO_3_5%	347.77	−1.305	419.17	16.01%

**Table 6:** Mechanical properties of TPU/MWCNT/BiFeO_3_ composites.

Samples	Tensile Strength (MPa)	Elongation at Break (%)	Tensile Toughness (MJ/m^3^)
TPU/MWCNT1%	12.80	349.10	32.40
TPU/MWCNT1%/BiFeO_3_1%	8.01	140.79	7.75
TPU/MWCNT1%/BiFeO_3_3%	5.60	66.20	2.09
TPU/MWCNT1%/BiFeO_3_5%	4.49	33.27	0.67

## Data Availability

The original contributions presented in this study are included in the article. Further inquiries can be directed to the corresponding authors.
